# Optimizing Single-Position Prone Lateral Lumbar Interbody Fusion with Exoscopic Technology: A Review of Key Innovations

**DOI:** 10.3390/jcm14041132

**Published:** 2025-02-10

**Authors:** Christian Quinones, John Preston Wilson, Deepak Kumbhare, Bharat Guthikonda, Stanley Hoang

**Affiliations:** Department of Neurosurgery, Louisiana State University Health Shreveport, Shreveport, LA 71103, USAjpw002@lsuhs.edu (J.P.W.);

**Keywords:** minimally invasive spine surgery (MISS), spinal fusion, prone transpsoas, single-position surgery, interbody fusion, lumbar stenosis, lateral, transpsoas

## Abstract

Minimally invasive spine surgery has advanced significantly over the past decade, integrating technologies such as intraoperative navigation, robotics, and artificial intelligence with innovative techniques such as single-position prone lateral transpsoas lumbar interbody fusion (proLIF). While proLIF offers excellent clinical outcomes for a wide range of lumbar pathologies, the lateral approach to lumbar spine presents technical and ergonomic challenges, including an increased need for soft-tissue dissection and unfavorable ergonomics for surgeons. This review details how the combination of emerging technologies has been applied in minimally invasive lumbar spine surgery. It also describes the novel application of an exoscope during navigation-guided proLIF. The benefits offered by the exoscope included high-resolution, three-dimensional visualization, enhanced maneuverability, and improved surgeon ergonomics. By combining emerging technologies with novel surgical approaches, this review demonstrates the recent advancements in minimally invasive spine surgery and underscores the exoscope’s potential to enhance visualization and optimize ergonomics for surgeons.

## 1. Introduction

Minimally invasive spine surgery (MISS) for the lumbar spine has evolved rapidly over the past decade, driven by the integration of enabling technologies such as intraoperative navigation, robotic platforms, and artificial intelligence. Historically, lumbar interbody fusion techniques such as the anterior (ALIF), posterior (PLIF), and transforaminal approaches involved narrow surgical corridors with limited visualization. The lateral lumbar interbody fusion (LLIF) emerged as an alternative surgical approach; however, it was still limited by either unnatural posterior hardware placement or the need for intraoperative patient repositioning. The refinement of surgical approaches, combined with emerging technological advancements, has led to the development of more efficient and less invasive techniques such as the single-position prone lateral transpsoas lumbar interbody fusion (proLIF). By integrating the advancements in intraoperative imaging, neuromonitoring, high-definition visualization tools such as exoscopes, this approach allows for simultaneous lateral interbody cage placement and posterior fixation in a single position, thus improving surgical efficiency. Moreover, with its ability it to address a wide range of lumbar pathologies while providing patients with excellent clinical outcomes, proLIF has expanded the scope of minimally invasive spine surgery (MISS) [1,2,3].

Despite these advantages, the proLIF approach has notable disadvantages, including the need for increased dissection and mobilization of vasculature when accessing distal lumbar segments, a heightened risk of peripheral nerve injury, and a reliance on neuromonitoring [4]. Additionally, the unfavorable ergonomics of the procedure, requiring the surgeon to adjust neck and body positions for optimal visualization, can lead to discomfort and workflow inefficiencies.

Exoscope-assisted spine surgery has emerged as a potential solution to some of the challenges associated with proLIF, such as enhanced visualization of the surgical field and improved surgeon ergonomics. The exoscope’s ability to provide a high-resolution, three-dimensional (3D) view of even the narrowest surgical corridor has been described as superior to other visualization techniques such as the operative microscope [5,6,7,8]. These advantages stem from the exoscope’s unique design, which offers enhanced mobility and maneuverability, significantly improving surgeon comfort without disturbing operative workflow or the sterility of the surgical field [6,9]. Consistent with the principles of MISS, studies of exoscope-assisted lumbar spine surgery have reported reduced operative times and shorter hospital stays [6,7,8].

Although they offer several benefits, exoscopes are not without their own set of unique limitations. For example, 3D visualization requires the user to wear a pair of 3D glasses which can cause discomfort and interfere with a surgeon’s operative loupes [6]. Additionally, while the learning curve for exoscopes is shorter than that of endoscopic techniques, its application to novel surgical approaches must be defined, practiced, and perfected. This challenge may explain the occasional reports of increased intraoperative blood loss during initial exoscope use in spine surgery [6,7,8]. Furthermore, the high costs of exoscopes limit their widespread adoption, especially in low-resource settings [6,7,8].

Overall, definitive conclusions regarding the use of exoscopes during spine surgery remain limited by the volume of available evidence. Existing studies lack long-term clinical outcome data, a definitive workflow for integration into specific procedures, and clear guidelines for operating room positioning. By addressing these deficiencies, future studies will aid in confirming and further defining the purported advantages of exoscopes in spine surgery. This review aims to examine the role of exoscopes in MISS with a particular focus on their integration into a navigation-guided proLIF. This paper evaluates the current literature for the impact of exoscope-assisted spine surgery on visualization, ergonomics, and surgical efficiency as well as the novel and future integration of recent advancements in enabling technologies. Additionally, we present the novel use of an exoscope to illustrate the feasibility, technique, and logistics of its integration during navigation-guided proLIF.

## 2. Advances in Minimally Invasive Spine Surgery

### 2.1. Enabling Technologies

**Figure 1:**
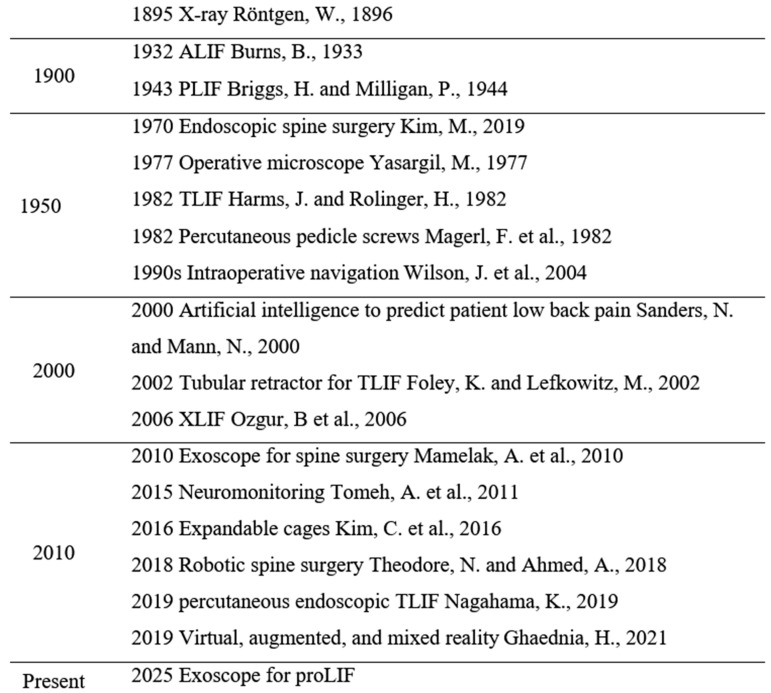
Enabling technology timeline for spine surgery [8,9,10,11,12,13,14,15,16,17,18,19,20,21,22,23,24,25]. Abbreviations: ALIF = anterior lumbar interbody fusion; PLIF = posterior lumbar interbody fusion; TLIF = transforaminal lumbar interbody fusion; XLIF = extreme lateral interbody fusion; proLIF = single-position prone lateral transpsoas lumbar interbody fusion.

Minimally invasive spine surgery (MISS) represents an effort to improve patient outcomes and surgical efficiency while reducing complications. Its development has been propelled by advances in surgical approaches, improved implantable hardware, sophisticated imaging, and the advent of intraoperative navigation, neuromonitoring, robotic systems, and the application of artificial intelligence (Figure 1). Emerging technologies such as virtual reality (VR), augmented reality (AR), and mixed reality (MR) have been described and will likely provide unique advantages to the world of MISS. Collectively, these enabling technologies represent milestones in the ongoing evolution of MISS.

The advancement of minimally invasive spine surgery has been propelled by technological innovations in surgical instruments such as the endoscope and tubular retractor system [10]. Similarly, the introduction of the operative microscope marked a major milestone in surgical visualization in spine surgery [11]. By the 1970s, its benefits were universally recognized within the field of spine surgery [12]. Endoscopes and operative microscopes, however, are not without limitations. For instance, the ergonomic constraints of operative microscopes often require surgeons to maintain uncomfortable head and neck positioning [11]. Endoscopy, on the other hand, offers a limited field of view and confined surgeons to a restricted working space [11].

Modern advances that have improved MISS include innovative surgical approaches and expandable cages [13]. The ALIF approach was first described by Burns in 1933 [14], while the PLIF and the TLIF [15] merged as foundational techniques in lumbar spine surgery during the 1990s. However, it was not until 2006 when Ozgur et al. described the lateral approach to lumbar interbody fusion, marking a pivotal turning point in lumbar fusion techniques and giving rise to several variations [16]. Lateral approaches in lumbar spine surgery align with the basic principles of MISS, including minimizing soft-tissue disruption, reducing complications, and hastening recovery [17].

Specifically, proLIF has gained popularity due to its versatile access to spinal pathology and favorable clinical outcomes [1,2,3]. The anatomic advantages of the prone position confer several distinct benefits, including decreased likelihood of visceral injury in a gravity dependent position [18] and minimal mobilization of the great vessels and sympathetic plexus [19]. This approach utilizes a corridor that allows for the placement of a larger interbody cage, facilitating the restoration of a significant degree of lumbar lordosis [20,21]. Additionally, performing the procedure in a single position eliminates the need for position changes, enabling simultaneous lateral interbody cage placement and posterior instrumentation [22,23,24]. The risk profile of proLIF is comparable to that of other lateral approaches, with sensory nerve injury and transient psoas weakness being the most common complications [21,25].

ProLIF utilizes a transpsoas approach, which inherently carries a risk of neurologic injury to the lumbar plexus. The development of intraoperative neuromonitoring (IONM) revolutionized the ability to monitor and potentially mitigate neurologic injury during such procedures. Electromyography was the first modality of IONM employed in the proLIF technique; since then, various other modalities have been described [26]. While several studies have advocated for the use of multimodal IONM, a universal consensus of its optimal application remains elusive [27]. Looking ahead, artificial intelligence (AI) holds promise for the development of improved and automated IONM systems, potentially enhancing surgical precision and patient safety [28].

Advancements in spine imaging have provided surgeons with the tools to more precisely delineate surgical anatomy. Enhanced visualization improves accuracy and enables less invasive surgical techniques. Traditional intraoperative imaging techniques, such as two-dimensional (2D) fluoroscopy, were first introduced in the 1990s. Subsequently, computed tomography (CT)-guided navigation emerged as a significant innovation. By analyzing preoperative and intraoperative imaging, CT-guided navigation software (StealthStation 2.1.0 (2.1.0-52)) displays localized surgical anatomy in real time, allowing for the precise visualization of instrument positioning relative to surgical structures. Compared to fluoroscopic or freehand techniques, CT-guided intraoperative navigation offers several advantages, including safer surgical approaches [29], increased pedicle crew placement accuracy, and reduced radiation exposure [29,30,31,32,33,34,35,36,37]. By 2010, intraoperative navigation using CT-guided imaging had achieved widespread adoption in spine surgery [37].

Robotic systems represent a transformative advancement in minimal invasive techniques. These systems minimize soft tissue damage and intraoperative blood loss, leading to reduced postoperative pain, shorter recovery times, and greater accuracy in screw placement compared to fluoroscopy-assisted techniques [35,36,38]. However, they are associated with increased operative times, higher upfront costs, and a steep learning curve for surgical teams [39,40]. The literature has consistently demonstrated the feasibility and accuracy of robotic technology in spine surgery. Robotic systems have been shown to slightly increase operative times and intraoperative blood loss while delivering greater accuracy compared to fluoroscopy-assisted spine surgeries [35,36,38]. These benefits are consistently observed across the most commonly used robotic platforms, demonstrating uniformly high accuracy, low complication rates, and comparable reoperation rates among four robotic platforms [38].

Robotic-assisted navigation in a single-position prone lateral transpsoas approach has been proven safe [33,41,42,43], with reported accuracy rates as high as 98% in single-position lumbar interbody fusions [39,41,44]. Feasibility studies further support the utility of this approach [41,45]. Additionally, learning curves for robotic systems have been objectively defined through metrics such as total operative time, screw placement time, and radiation dose [39,40]. One way in which studies have quantified the learning curve for these systems is by measuring these metrics across cases, and, once stabilized, proficiency is reached [40]. One study reports significant improvements after only 13 cases [46].

### 2.2. Application of the Exoscope in Single-Position Prone Lateral Lumbar Fusion

**Figure 2:**
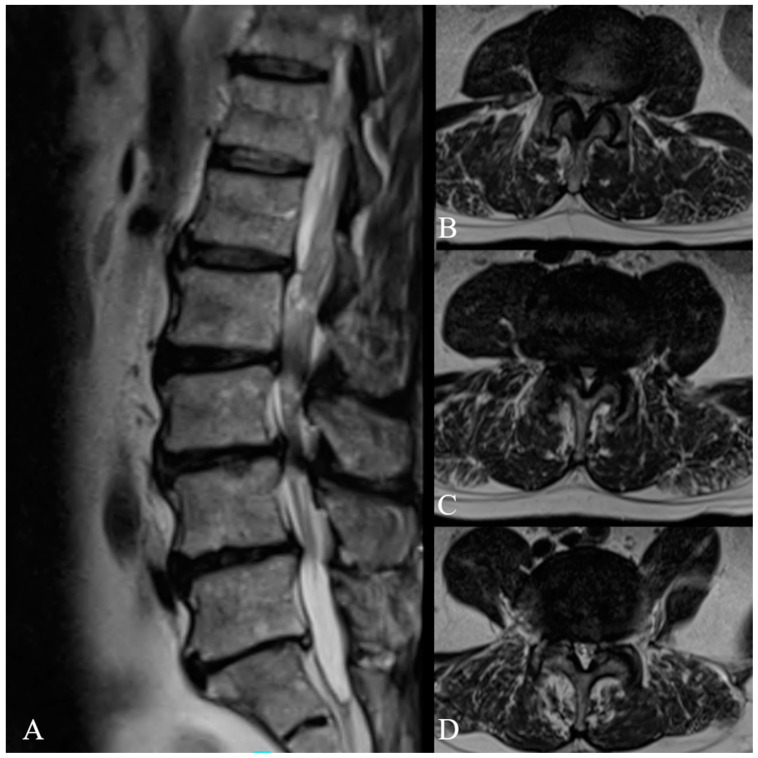
Preoperative MRI demonstrating: (**A**) multilevel central stenosis, (**B**) central and bilateral foraminal stenosis at L2-3, (**C**) central and bilateral foraminal stenosis at L3-4, (**D**) central and bilateral foraminal stenosis at L4-5.

A 66-year-old male with a history of diabetes mellitus and hypertension presented with a long-standing history of mechanical back pain and bilateral radiculopathy. His symptoms continued to progress despite several months of conservative therapy. Physical exam revealed full strength but diminished sensation. Magnetic resonance imaging (MRI) revealed L2-5 central and bilateral foraminal stenosis (Figure 2). Having failed conservative management, the patient desired to pursue surgical intervention, and he was offered a minimally invasive lateral lumbar interbody fusion at L3-4 with posterior decompression and fusion from L2-5.

**Figure 3:**
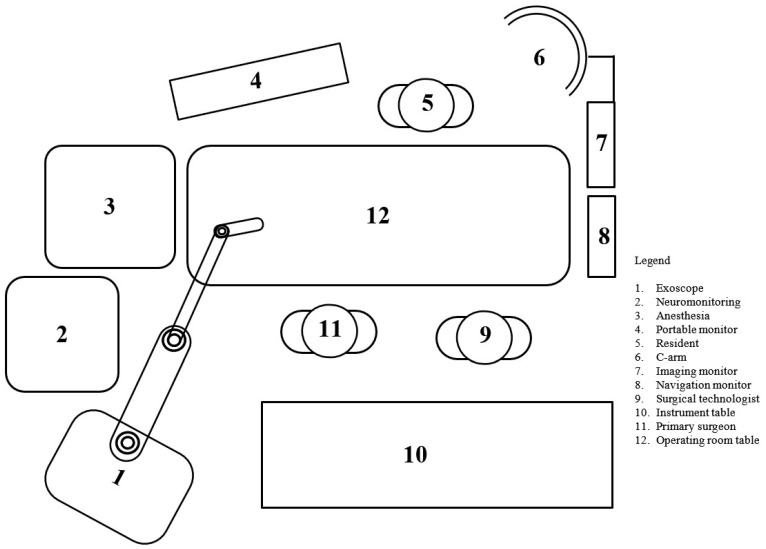
Schematic representation operating room setup. Legend: 1. exoscope; 2. neuromonitoring; 3. anesthesia; 4. portable monitor; 5. resident surgeon; 6. C-arm; 7. imaging monitor; 8. navigation monitor; 9. surgical technologist; 10. instrument table; 11. primary surgeon; 12. operating room table.

**Figure 4:**
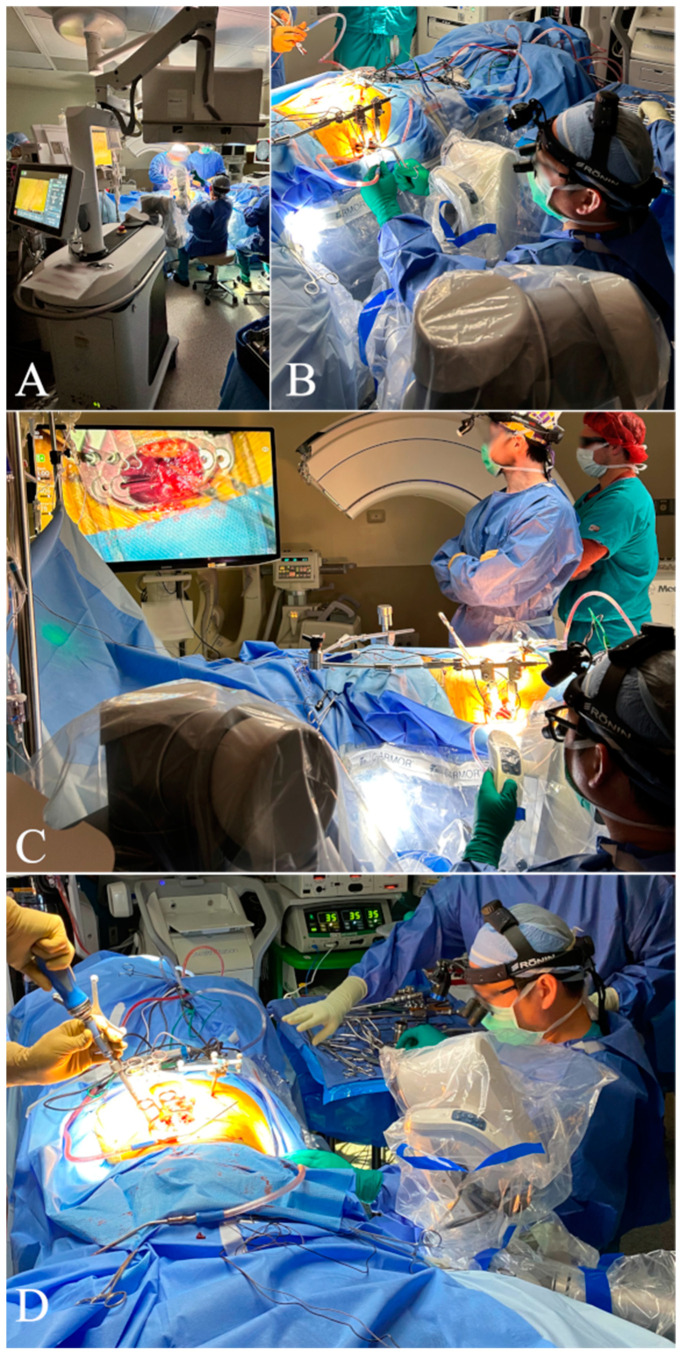
Operating room setup: (**A**) exoscope positioned behind and to the left of the surgeon, (**B**) close-up view of the exoscope location, (**C**) surgeon viewing the monitor with a neutral line of sight, (**D**) exoscope repositioned away from the surgical site.

The equipment required for this procedure included: intraoperative CT, navigation system with monitor, fluoroscopy (C-arm), associated instruments, neuromonitoring, exoscope, and lateral retractors. The exoscope was placed behind and to the left of the primary surgeon, as shown in the schematic (Figure 3) and operating room setup (Figure 3 and Figure 4).

The patient was placed in the prone position on a Jackson table, and hip pads were adjusted to maximize ipsilateral surgical site access and contralateral support. Tape was used to secure the patient to the bed. Next, incision sites for posterior and lateral incisions were marked. The surgical field was then draped and prepped in the usual sterile manner. A percutaneous iliac pin was placed in the posterior superior iliac spine, and a reference frame was attached. The Medtronic O arm was positioned over the surgical field, and the acquired images were uploaded into the navigational system. The operative plan was confirmed by the attending surgeon, and final registration was conducted and verified by assessing the accuracy of the pointer device over specific bony landmarks.

**Figure 5:**
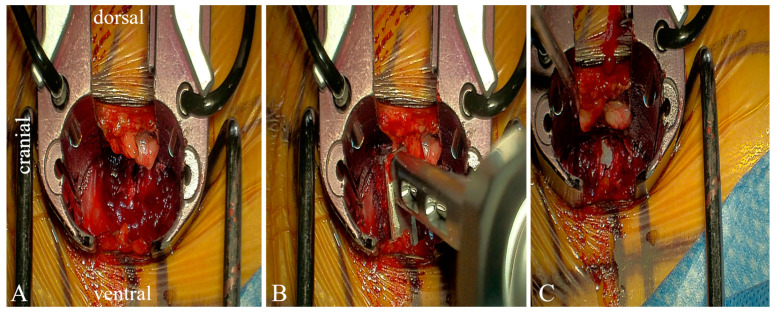
Intraoperative exoscope footage through retractor system: (**A**) partial discectomy, (**B**) cage being introduced, (**C**) after cage has been implanted. In all figures, the left side is cranial, right side is caudal, top is dorsal, and bottom is ventral.

The lateral approach began with left abdominal skin incision followed by blunt dissection through the subcutaneous fat, oblique muscles, and transversalis muscle until the retroperitoneal space was reached. A navigation probe was passed through this retroperitoneal corridor, through the psoas muscles and into the disk space. Neurostimulation was applied to sequential dilators as they were subsequently passed through the psoas muscle. The retractor was placed over the dilators and attached to the bed frame. An annulotomy was performed, and a combination of distractors and trials were used to prepare the disk space (Figure 5).

The exoscope’s built-in monitor was initially positioned with the assistance of an operating room staff member. Once it was approximately aligned over the lateral portion of the planned surgical field, the surgeon made further fine-tuned adjustments to optimize visualization. To achieve the best possible alignment, the surgeon evaluated multiple angles and distances, making incremental modifications until the optimal field of view was established. Proper placement was essential for maintaining an unobstructed view while preserving flexibility in repositioning the device throughout the procedure. Throughout this process, careful attention was given to ensuring that the exoscope’s placement did not encroach upon the surgeon’s working area or interfere with surgical instruments, anesthesia monitoring, or sterile precautions. After achieving the desired positioning, the staff confirmed that the exoscope could be placed and removed from the surgical field without interfering with the primary surgeon, anesthesia staff, or compromising sterility. This setup enabled seamless transitions between different phases of the operation, reducing disruptions and maintaining consistent image quality. The second, mobile exoscope monitor was placed opposite the primary surgeon, ensuring an unobstructed and ergonomically favorable viewing angle, which allowed the surgical team to maintain an interrupted workflow while benefiting from high-resolution visualization.

**Figure 6:**
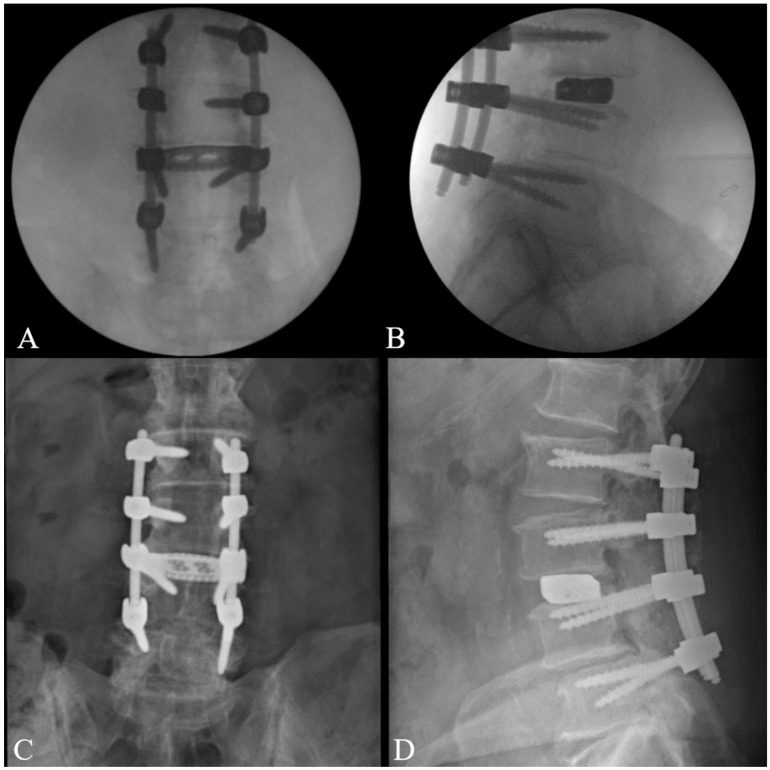
Intraoperative fluoroscopy: (**A**) AP and (**B**) lateral. Six-week postoperative imaging: (**C**) AP and (**D**) lateral.

The posterior decompression and fusion were performed simultaneously with the lateral stage of the procedure. Briefly, bilateral L2-5 pedicle screws were placed under intraoperative guidance. This was followed by bilateral L2-5 laminectomies, foraminotomies, and lateral recess decompression. Rods were placed, and set screws were tightened. Intraoperative films confirmed the proper placement of pedicle screws and rods (Figure 6). The surgical site was inspected, irrigated, and closed. The patient’s post-op neurologic exam was without concern for neurologic complications, and they were discharged home on post-operative day 2. At the six-week follow-up evaluation, the patient reported recovering well, with postoperative imaging confirming the proper positioning of the hardware (Figure 6).

## 3. Discussion

### 3.1. Exoscope Experience

The enhanced maneuverability and ergonomics of exoscopes results from their intuitive controls, which allow for quick, minor adjustments during surgery, ensuring procedural continuity [47]. The exoscope’s articulated arms provide multiple degrees of freedom, enabling the horizontal, vertical, and axial adjustment for the optimal visualization of the surgical field. The arm’s range of motion allows it to extend and retract, facilitating entry and exit from the surgical field without disrupting staff or equipment. The exoscope’s movement is manually controlled by the surgeon, surgical assistant, or other surgical staff member at the base. Additionally, its head offers a high degree of rotational movement, enabling precise adjustments. Once positioned, a locking system secures the view, and the position can be saved for temporary removal and accurate repositioning. This flexibility enhances the visualization of anatomy, surgical corridors, and instruments used during spine surgeries [8].

The exoscope also features an exceptional stereotactic display that enables non-coaxial interaction [48]. Unlike traditional microscopes, which require both eyes to align with the optical path, the exoscope allows users to view the same high-quality display from various positions. During the procedure, the exoscope was placed behind and to the left of the primary surgeon, adjacent to the anesthesia team. This placement maintained the base at a safe distance from the sterile field, preventing interference with the passing of surgical instruments, as described in other studies [49]. The case was displayed on two monitors: larger, free-standing 3D monitor opposite the primary surgeon and a smaller monitor attached to the exoscope base. If required, the larger monitor could be repositioned to accommodate imaging equipment. This setup avoided spatial limitations typically encountered in similar operative scenarios [49].

The exoscope was used to visualize essential anatomical landmarks during the initial exposure and disk preparation phase. The lateral approach was performed from a sitting position, with the exoscope entering and exiting the surgical field from the surgeon’s left. Its high-definition magnification provided enhanced clarity of soft tissue and vascular anatomy, allowing for meticulous dissection and the preparation of the intervertebral space. The automated locking system played a key role in maintaining workflow efficiency. By engaging this system, the exoscope could be smoothly repositioned as needed and returned to its prior viewing position without requiring constant manual adjustments. This functionality was particularly beneficial during key procedural steps, such as cage placement and endplate preparation, ensuring that the surgeon could seamlessly transition between direct visualization and exoscopic-assisted views. Additionally, the ability to retract the exoscope without disrupting the operative field or requiring frequent assistance from staff contributed to improved surgical ergonomics and reduced operative time. The ergonomic design allowed the surgeon to maintain a consistent chair height and neutral line of sight, eliminating the need to adjust the operating table’s height or angle. These ergonomic advantages align with previous studies that report improved surgeon comfort during exoscope-assisted spine surgery [5,6,47,50,51]. The learning curve for exoscopes in spinal procedures has been described as straightforward [49]. This ease of use is attributed to the exoscope’s similarity to traditional operative microscopes and its adjustability by the surgeon, technician, or resident, which facilitates smoother integration into the surgical workflow [49].

The integration of enabling technologies has propelled the field of MISS. Each advancement, no matter how incremental, serves as a steppingstone in the evolution of this field. In the case described, the patient achieved excellent postoperative outcomes without adverse events, consistent with findings from a literature review of exoscopes in spine surgery by Vatipally et al. [8]. Building upon these advancements, future innovations in MISS are expected to further refine surgical precision, improve patient outcomes, and expand the scope of what is achievable through technologies such as artificial intelligence, robotics, and advanced imaging modalities.

### 3.2. Future Advancements

As healthcare systems increasingly prioritize value-based care, it is essential to consider the monetary and nonmonetary costs associated with enabling technologies for MISS. With a greater emphasis on healthcare value, it is vital to consider the monetary cost to hospitals, patients, and surgeons. Previous studies suggest that exoscopes may be less costly than operating microscopes in spine surgery [52], although this can vary depending on the specific model chosen [8]. Nonmonetary factors, such as reoperation rates, operative times, and radiation exposure, also influence the overall cost-effectiveness of exoscopes, navigation systems, and robotic platforms. Comprehensive studies are needed to accurately evaluate the economic impact of these technologies in the advancement of MISS [53].

One significant barrier to the adoption of robotic and navigational systems is their high cost, particularly for early-career surgeons [54]. However, some studies support their economic feasibility by demonstrating reductions in revision surgery rates [33,55]. Despite these findings, there exist limitations to accurately analyzing the cost of spinal fusion procedures. For example, the reliance on Medicare reimbursement data categorized by diagnosis-related groups does not account for the heterogeneity of procedures or patient-specific factors [56]. By staying up to date with technological advancements capable of improving surgical ergonomics and longevity, surgeons will be better prepared to address future challenges such as the physician shortage [7].

A limiting factor in the incorporation of novel technologies in spine surgery is spatial constraints within the operating room. The development of exoscopes specifically designed for spine surgery can facilitate easier incorporation into the surgical field [8]. Additionally, operating rooms tailored specially to spine surgery can optimize the available space. Studies suggested that integrating monitors for imaging and navigation into the operating room enhances space utilization and improve workflow ergonomics [33,42]. Customized operating rooms can also improve image acquisition by accommodating advanced equipment, incorporating tables with increased range of motion, and integrating navigational and registration cameras compatible with augmented reality systems [29,33,42,53].

Advancements in imaging technology have the potential to improve visualization during spine surgery by enhancing imaging quality, reducing radiation exposure, increasing compatibility with intraoperative navigation and robotics, and integrating virtual and augmented reality systems. For intraoperative navigation, future imaging systems are expected to enable faster acquisition and employ more compact equipment [33]. Navigation registration will also become more efficient, incorporating multiple imaging modalities to enhance accuracy [33]. With greater imaging precision and optimized intraoperative workflows, overall radiation exposure is expected to decrease [29,33]. The incorporation of MRI for navigation along with coregistration capabilities has the potential to further improve intraoperative imaging. For example, some studies demonstrated the feasibility of eliminating preoperative CT imaging for navigation by utilizing MRI instead, which would improve soft tissue localization and dissection [42,57]. In addition to the integration and coregistration with MRI, the use of intraoperative ultrasound for neuronavigation has demonstrated feasibility in preliminary animal studies [58].

Artificial intelligence has influenced multiple aspects of MISS. For instance, one study demonstrated AI’s capability to generate CT scans from MRI [59], while another study showcased an AI system capable of performing autonomous, robotic cadaveric posterior decompression [60]. AI also holds promise for enhancing outcome predictions, improving research efficiency, and optimizing perioperative data tracking, intraoperative navigation, and postoperative complication prediction [61,62,63]. The incorporation of AI and other enabling technologies to robotic systems will also further improve current advantages of robotic systems such as hardware placement accuracy [61] and reduced intraoperative radiation exposure. However, the reduction in radiation exposure requires further investigation, as significant variation has been reported across studies, likely due to differences in scanning protocols, surgical procedures, and the metrics used to report outcomes [38]. With continued innovation in AI and robotic systems, the possibility of telesurgery systems is anticipated in the near future [64]. Studies have begun to lay the groundwork for telesurgery by incorporating AI into robotic system to provide haptic feedback during surgery [65]. This advancement provides surgeons with a tactile sense of the forces being applied during surgery [65]. This will allow real-time sensory input to enhance precision, reduce the risk of tissue damage and improve safety [65]. Currently, however, haptic feedback from robotic systems still requires a great deal of development prior to integration into everyday practice [64].

The advancement of enabling technologies will not only enhance surgical performance but also revolutionize the training of surgical residents. As previously discussed, exoscopes provide superior visualization of surgical procedure, however, other technologies contribute to this as well. For instance, the use of 3D footage has been demonstrated to improve the conceptualization of surgical techniques during training. One study comparing 2D and 3D simulation training for lumbar disk herniation found 3D simulation to be significantly more effective in improving objective and subjective performance [66]. Enhanced visualization technologies also enable the integration of virtual, augmented, and mixed reality in spine surgery [67,68]. Virtual reality can create an immersive digital 3D environment that is experienced through head-mounted displays., Augmented reality can project images onto physical objects, and mixed reality combines a fully digital experience as well as haptic feedback [69]. Studies have already demonstrated objective and subjective improvements in pedicle screw placement during surgical training using these technologies [67,69,70]. At the crossroads of improved visualization, AI, and surgical training lies video-based automated performance metrics. While annotating surgical footage is traditionally time-consuming, AI offers the potential to streamline this process through automation. One study used AI to analyze surgical video recordings, predict surgeon characteristics, assess performance, and provide recommendations for improvement [71].

By harnessing innovation and rapid technological advancement, MISS can further increase patient safety, outcomes, and operative advantages. The combination of exoscope technology with AI and robotics represents an exciting frontier in the evolution of MISS. AI-powered exoscopes may one day automatically adjust focus and decrease the frequency of microscope adjustments, thereby increasing operative efficiency. Moreover, this could be extended to analyze surgical procedure steps and performance and provide surgeons with an individualized breakdown of surgical efficiency and areas of improvement, which would also benefit resident training by providing a means of objective evaluation and education simulations. Furthermore, AI-powered exoscopes could integrate with robotic systems and imaging studies to improve intraoperative navigation, enhance surgical planning, and increase hardware placement accuracy and precision. Despite these promising advances, there remains a host of challenges, including the computational requirements of real-time AI processing, limited high-quality datasets, and monetary considerations. In combination with these challenges, there are several limitations to the exemplary use of the exoscope for proLIF. First, application of this technology described a single case, limiting the generalizability of findings. Second, the specific details regarding the exoscope model and its magnification settings were not recorded, which prevented direct comparisons with other visual systems. Third, the cost of the exoscope was not disclosed. Fourth, there is no quantitative analysis of surgeon ergonomics, which would be required to accurately assess the potential ergonomic benefits of exoscope-assisted proLIF. These limitations highlight the need to conduct studies with larger sample sizes at multiple centers to determine the impact that exoscope use in spine surgery.

## 4. Conclusions

This review details the history of enabling technologies that influenced the evolution of MISS. It also provides an example of how these advancements can be combined and applied to novel surgical approaches, giving rise to proLIF. To our knowledge, this is the first reported case describing the use of an exoscope to assist in a navigation-guided proLIF. The advantages observed include enhanced visualization of the surgical field, high maneuverability, improved surgeon ergonomics, and comfort. These findings support the exoscope as a viable adjunct in MISS. The combination of the rapidly advancing enabling technologies will propel the world of MISS. However, additional research is necessary to evaluate the long-term impact of exoscope use and other enabling technologies on clinical outcomes, operative efficiency, and surgeon performance.

## Abbreviations

The following abbreviations are used in this manuscript:

proLIF	Single-position prone lateral transpsoas lumbar interbody fusion
MISS	Minimally invasive spine surgery
VR	Virtual reality
AR	Augmented reality
ALIF	Anterior lumbar interbody fusion
PLIF	Posterior lumbar interbody fusion
TLIF	Transforaminal lumbar interbody fusion
XLIF	Extreme lumbar interbody fusion
IONM	Intraoperative neuromonitoring
AI	Artificial intelligence
NeM	Neuromonitoring
NvM	Navigation monitor
CT	Computed tomography
MRI	Magnetic resonance imaging
